# The 2022 International Society for Hip Preservation (ISHA) physiotherapy agreement on assessment and treatment of greater trochanteric pain syndrome (GTPS): an international consensus statement

**DOI:** 10.1093/jhps/hnac050

**Published:** 2023-01-02

**Authors:** Ashley Disantis, Antonio J Andrade, Alexander Baillou, Nicolas Bonin, Thomas Byrd, Ashley Campbell, Benjamin Domb, Holly Doyle, Keelan Enseki, Barry Getz, Lucie Gosling, Louise Grant, Victor M. Ilizaliturri Jr., Dave Kohlrieser, Jovan Laskovski, Liran Lifshitz, Ryan P. McGovern, Katie Monnington, John O’Donnell, Amir Takla, Tim Tyler, Mike Voight, Thomas Wuerz, RobRoy L Martin

**Affiliations:** Adolescent and Young Adult Hip Preservation Program, UPMC Children’s Hospital of Pittsburgh, 4401 Penn Ave, Pittsburgh, PA 15224, USA; Department of Physical Therapy, Rangos School of Health Sciences, Duquesne University, 600 Forbes Ave, Pittsburgh, PA 15282, USA; Reading Orthopaedic Centre, Circle Reading Hospital, Reading RG2 0NE, UK; Trauma and Orthopaedic Department, Royal Berkshire NHS Foundation Trust, Reading RG1 5AN, UK; Physiotherapy, Physio-Baillou,Praterstrasse, 60/1/3, A-1020, Vienna, AT; Orthopaedic Surgery, Lyon Ortho Clinic, 29B Avenue des Sources, Lyon 69009, FR; Orthopaedic Surgery, Nashville Sports Medicine Foundation, 100, 2011 Church Street, Nashville, TN 37203, USA; Physical Therapy, Performance One Physical Therapy and Wellness, 400 Franklin Road, Franklin TN 37069, USA; Orthopaedic Surgery, American Hip Institute, 999 E Touhy, Des Plaines, Chicago IL 60018, USA; Integrum Physiotherapy, 94 Ridge Rd, London N8 9NR, UK; Centers for Rehab Services/University of Pittsburgh Medical Center, Rooney Sports Complex, 3200 S. Water St, Pittsburgh, PA 15203, USA; Physiotherapy, The Centre for Sports Medicine and Orthopaedics, 9 Sturdee Ave, Johannesburg, Rosebank 2196, SA; Young Adult Hip Service, The Royal National Orthopaedic Hospital, 519 Briston Rd S, Birmingham B31 2AP, UK; Physiotherapy, PhysioCure, Cookridge Lane, Leeds S16 7NL, UK; Adult Joint Reconstruction, National Rehabilitation Institute of Mexico, Calz Mexico-Xochimilco 289, Coapa, Guadalupe Tlalpan, Tlalpan, 14389 Cuidad de Mexico, CDMS, MX; Physiotherapy, Orthopedic One, 4605 Sawmill Road, Columbus OH 43220, USA; Orthopedic Surgery, Crystal Clinic Orthopedic Center, Hip Preservation, 1622 SR 619, Ste 200, Akron, OH, USA; Physiotherapy, Physio & More, 27 Shabtai Yaacov, Tel Aviv- Yafo 6962806, IL; Sports Medicine Research, Texas Health Orthopedic Specialists, 6301 Harris Parkway, #200 Dallas/Fort Worth, TX 76132, USA; Young Adult Hip Service, The Royal National Orthopaedic Hospital, 519 Briston Rd S, Birmingham B31 2AP, UK; Hip Arthroscopy Australia, 21 Erin Street, Richmond VIC 3121, AU; Orthopaedics, St. Vincent’s Melbourne, East Melbourne, VIC 3065, AU; Hip Arthroscopy Australia, 21 Erin Street, Richmond VIC 3121, AU; Swinburne University of Technology, Hawthorn Campus, John Street, Hawthorn, Victoria 3122, AS; Australian Sports Physiotherapy, Ivanhoe 3079, Australia; Physiotherapy, NISMAT, 130 E 77th St, New York, NY 10075, USA; Professional Physical Therapy, New York, NY 10010, USA; Physical Therapy, Performance One Physical Therapy and Wellness, 400 Franklin Road, Franklin TN 37069, USA; School of Physical Therapy, Belmont University, 1900 Belmont Boulevard, Nashville, TN, US; Orthopaedic Surgery, New England Baptist Hospital, 40 Allied Drive, Dedham, MA 02026, USA; Department of Physical Therapy, Rangos School of Health Sciences, Duquesne University, 600 Forbes Ave, Pittsburgh, PA 15282, USA; Centers for Rehab Services/University of Pittsburgh Medical Center, Rooney Sports Complex, 3200 S. Water St, Pittsburgh, PA 15203, USA

## Abstract

The 2022 International Society of Hip Preservation (ISHA) physiotherapy agreement on assessment and treatment of greater trochanteric pain syndrome (GTPS) was intended to present a physiotherapy consensus on the assessment and surgical and non-surgical physiotherapy management of patients with GTPS. The panel consisted of 15 physiotherapists and eight orthopaedic surgeons. Currently, there is a lack of high-quality literature supporting non-operative and operative physiotherapy management. Therefore, a group of physiotherapists who specialize in the treatment of non-arthritic hip pathology created this consensus statement regarding physiotherapy management of GTPS. The consensus was conducted using a modified Delphi technique to guide physiotherapy-related decisions according to the current knowledge and expertise regarding the following: (i) evaluation of GTPS, (ii) non-surgical physiotherapy management, (iii) use of corticosteroids and orthobiologics and (iv) surgical indications and post-operative physiotherapy management.

## INTRODUCTION

Greater trochanteric pain syndrome (GTPS) encompasses multiple diagnoses including external snapping hip, trochanteric bursitis and gluteus medius (GMed) and gluteus minimis (GMin) tendinopathy or tearing [1, 2]. GTPS mainly affects women in their fourth to sixth decade of life, presenting with pain and tenderness in the lateral hip, which worsens with walking, stair climbing and/ or lying on the affected side [3, 4]. While the symptoms associated with GTPS were previously thought to be a result of trochanteric bursitis, gluteal tendinopathy with or without bursitis has been identified as the primary source of pain and dysfunction [5–8]. Bicket et al. [9] reported an incidence of GTPS of 3.29 patients per 1000 per year, making it more common than Achilles tendinopathy. Management of GTPS may include physiotherapy, injections, therapeutic modalities and/or surgical management [4, 10]. Despite its prevalence, there is a paucity of high-quality research investigating both non-operative and operative physiotherapy management. The purpose of the modified Delphi study was to present an international physiotherapy consensus statement to guide physiotherapy-related decisions according to the current knowledge and expertise regarding the following content areas: (i) evaluation of GTPS, (ii) non-surgical physiotherapy management, (iii) use of corticosteroids (CSIs) and orthobiologics and (iv) surgical indications and postoperative physiotherapy management.

## MATERIALS AND METHODS

### Study participants

Participants for the panel were selected from the International Society for Hip Preservation (ISHA) to represent experts in the management of GTPS. Specifically, physiotherapy ISHA members with expertise in surgical and non-surgical management of individuals with GTPS were identified by the senior author. In addition, surgeons with expertise in the management of individuals with GTPS were also identified. Of the 24 identified individuals, 100% agreed to participate. The panel consisted of 16 physiotherapists and eight orthopaedic surgeons representing seven countries and with an average of 21 years (range: 8–39 years) experience. A summary of the attributes of the panelists is presented in Table I.

**Table I.** A summary of panelist attributes

*Orthopaedic surgeons*							
*Name*	*Years of experience*	*Specialty*	*Number of hip-specific consultations per week*	*Hip surgical procedures per year*	*Academic post (Y/N)*	*Actively involved in research (Y/N)*	*ISHA member (Y/N)*
Antonio J. Andrade	20	Orthopaedic sports medicine, trauma	40	400	N	Y	Y
Nicolas Bonin	20	Hip arthroplasty and hip preservation	40	600	N	Y	Y
Thomas Byrd	32	Orthopaedic sports medicine	20	400	Y	Y	Y
Benjamin Domb	15	Hip preservation	60	800	Y	Y	Y
Victor M. Ilizaliturri	22	Orthopaedic surgery, hip and knee reconstruction, hip preservation	60	350	Y	Y	Y
Jovan Laskovski	10	Orthopaedic sports medicine	75	550	N	Y	Y
John O’Donnell	28	Orthopaedics and sports medicine	60	600	Y	Y	Y
Thomas Wuerz	9	Orthopaedic sports medicine	75	330	Y	Y	Y
*Physiotherapists*							
*Name*	*Years of experience*	*Specialty (ortho, sports, trauma)*	*Number of hip-specific consultations per week*	*Number of hip-specific physiotherapy patients per year*	*Academic post (Y/N)*	*Actively involved in research (Y/N)*	*ISHA member (Y/N)*
Alexander Baillou	21	Sports and orthopaedics	35	200	N	Y	Y
Ashley Campbell	11	Sports and orthopaedics	30	250	Y	Y	Y
Ashley Disantis	8	Sports and orthopaedics	25	150	N	Y	Y
Holly Doyle	15	Sports and orthopaedics	20	100	N	Y	Y
Keelan Enseki	20	Sports and orthopaedics	10	100	Y	Y	Y
Barry Getz	22	Sports and orthopaedics	25	180	N	Y	Y
Lucie Gosling	21	Musculoskeletal and orthopaedics	25	100	N	Y	Y
Louise Grant	30	Sports and orthopaedics	50	200	N	Y	Y
Dave Kohlrieser	14	Sports and orthopaedics	40	300	N	Y	Y
Liran Lifschitz	27	Sports and orthopaedics	30	180	Y	Y	Y
Ryan McGovern	12	Sports and orthopaedics	25		N	Y	Y
RobRoy Martin	29	Sports and orthopaedics			Y	Y	Y
Katie Monnington	23	Musculoskeletal and Orthopedics	20	150	Y	Y	Y
Amir Takla	24	Hip and pelvis, sports injuries	40	300	Y	Y	Y
Tim Tyler	30	Sports and orthopaedics	11	56	N	Y	Y
Mike Voight	39	Sports and orthopaedics	10	120	Y	Y	Y

### Study design

This study used a modified Delphi technique to efficiently structure group opinion over multiple survey rounds [11, 12]. The three-step modified Delphi technique is recommended for use in healthcare to determine consensus for a clinician problem [13, 14]. This modified Delphi study allowed for focused discussion and judgement to be made on questions related to the assessment and treatment of GTPS. Methods for this study are similar to those described by Takla et al. [15]; briefly, the panel generated a list of relevant questions regarding physiotherapy management of GTPS. Consensus was then reached over three survey rounds. *A priori*, consensus was defined as 80% agreement.

## RESULTS

The panel determined that the following questions were to be answered:

- What is the definition of GTPS?
- What are the differential diagnoses that should be considered during the evaluation of a patient with suspected GTPS?
- What is the appropriate physiotherapy management of GTPS?
- Should pharmacologic agents be utilized in the setting of GTPS?
- Should physiotherapy be utilized following the use of CSIs or orthobiologics?
- What are the indications for surgical management of GTPS?
- Are there prognostic indicators that would negatively affect outcomes following the surgical management of GTPS?
- Should physiotherapy be utilized following the surgical management of GTPS?

Consensus was achieved for 7/8 questions. Panelist consensus was not achieved for the indications to surgical management of GTPS. Answers to the above questions are presented in the discussion.

## DISCUSSION

### What is the definition of GTPS?

*Consensus statement*: The term GTPS encompasses multiple diagnoses, including external snapping hip (coxa sultans), proximal iliotibial band syndrome, trochanteric bursitis, and GMed and/or GMin tendinopathy or tearing.

The term GTPS encompasses multiple diagnoses including external snapping hip (coxa sultans), also known as proximal iliotibial band syndrome, trochanteric bursitis and GMed and/or GMin tendinopathy or tearing [1, 2]. The symptoms of GTPS were previously thought to be a result of trochanteric bursitis; however, the term bursitis implies findings consistent with inflammation, which have not been demonstrated in the literature. Instead, multiple histologic and imaging studies have identified gluteal tendinopathy with or without bursitis as the primary source of pain and dysfunction. No histological differences of the trochanteric bursa have been identified between individuals with GTPS and healthy controls [5–8]. In a study of 24 individuals with clinical features of GTPS, Bird et al. [6] reported that 8.3% of individuals had magnetic resonance imaging (MRI) findings consistent with trochanteric bursal distention, while 45.8% and 62.5% had findings consistent with a GMed tear and GMed tendinitis, respectively. Similarly, ultrasound evaluation of 877 individuals with GTPS found no signs of bursitis in 79.8% of individuals, while 49.9% and 28.5% had signs of gluteal tendinopathy and thickening of the iliotibial band, respectively [16]. Therefore, the term GTPS is non-specific and refers to pain and dysfunction of the many aforementioned structures in the lateral hip. This is similar to the use of the term ‘non-specific low back pain’, which encompasses many different pathologies including but not limited to muscular strains, spinal stenosis and intervertebral disc degeneration [2].

### What are the differential diagnoses that should be considered during the evaluation of a patient with suspected GTPS?

***Consensus statement*** **: When evaluating GTPS, differential diagnoses should include intra-articular hip disorders, lumbosacral spine disorders, and muscle-tendon disorders of the hip.**

Individuals suffering from GTPS complain of pain at the lateral hip, with symptoms potentially radiating to the level of the buttock and lateral thigh. Pain generally worsens with single limb weight-bearing activities and lying on the affected side. The greatest degree of tenderness should specifically be located on the lateral or posterior aspect of the greater trochanter [5, 8, 17, 18]. Activities that exacerbate symptoms include walking and ascending stairs, as well as other single leg activities, such as putting on pants or getting in/out of the bathtub.

Due to the high likelihood of overlapping pathologies, particularly hip joint osteoarthritis and low back pain, it is important to first assess for the presence of intra-articular hip and/or lumbar spine pathology. Individuals complaining of radiating pain below the knee, pain in the low back that increases with sitting or walking, and pain specifically associated with movement of the lumbar spine should undergo a comprehensive lower quarter screen. This examination should assess for myotome-related weakness, decreased sensation in dermatomal pattern, abnormal findings during lower extremity reflex testing, and lumbar range of motion. For those with groin pain and morning stiffness, hip osteoarthritis should be considered [19]. Intra-articular hip joint pathology should be considered with the reproduction of groin pain during a flexion-abduction-external rotation (FABER) test, flexion-adduction-internal rotation, internal rotation range of motion with overpressure (IROP) and/or Scour test, decreased range of motion in a capsular pattern and demonstration of an antalgic gait pattern [15]. Those presenting with pain localized to the posterior thigh with tenderness at the ischial tuberosity or pain localized to the posterior hip with tenderness lateral to the ischial tuberosity should be evaluated for hamstring or deep gluteal syndrome, respectively [20].

### What is the appropriate physiotherapy management of GTPS?

***Consensus statement*** **: Physiotherapy management of GTPS should be impairment based targeting the deficits identified during the physical examination.**

Currently, there is no test or cluster of tests utilized to identify which *specific* pathoanatomical structures are involved or the extent to which they are involved. Commonly utilized special tests to evaluate for the presence of GTPS include resisted hip abduction and external rotation test, FABER test, resisted external de-rotational test and Trendelenburg test [6, 21, 22]. Ganderton et al. [21] found the highest diagnostic accuracy in confirming GTPS with the FABER test, resisted hip abduction, and the resisted external de-rotational test. It should be noted, however, that MRI evaluation found gluteal pathology in 88% of these individuals, ranging from mild tendinosis to a full thickness tear [21]. Sensitivity and specificity values of 73% and 77% and 88% and 93.7% for the Trendelenburg test and the resisted external rotation test, respectively, have been previously reported in individuals with GTPS [6, 23]. Along with special tests, palpation of the greater trochanter and trochanteric bursae may be a useful tool when evaluating for GTPS [7, 21, 24]. In a study of individuals with lateral hip pain, Grimaldi et al. [7] reported that those who were not tender on palpation over the greater trochanter were unlikely to have gluteal tendinopathy on MRI.

Besides confirming the presence or absence of GTPS, these special tests offer little to the clinician in prescribing appropriate treatment interventions. Therefore, clinicians should evaluate for impairments related to the development of GTPS. Factors associated with the development of GTPS include hip abductor weakness, loss of pelvic control in the frontal plane and iliotibial band tightness and thickening as they increase the compressive forces through the bursae and GMed and GMin tendons [25, 26]. Sutter et al. [27] found hypertrophy of the tensor fascia lata secondary to overcompensation in individuals with long-standing GMed and GMin insufficiency. Strategies to improve hip abductor strengthening while avoiding overactivity of the tensor fascia lata should be utilized [28]. Disantis et al. [29] presented an impairment-based treatment classification system for patients with GTPS, classifying individuals based upon the presence of non-contractile or contractile findings and further subclassifying based upon irritability. Strengthening exercises are progressed from submaximal isometrics to heavy load and eccentric exercises based on patient tolerance [29]. In addition to improving strength and tendon healing, exercise may also play an analgesic role possibly through the central nervous system and reduced cortical inhibition [30].

Along with a tailored exercise program, extensive education should be provided regarding activity and postural modifications with the goal of reducing load and compression through the lateral hip. Strategies should be given to help minimize time spent on a single leg during activities of daily living, such as sitting rather than standing to put on trousers, thereby reducing tensile load through the gluteal muscles. The importance of avoiding hip adduction should be stressed for sitting, standing and sleeping to limit excessive compression through the lateral hip [31]. For example, education should be provided to avoid crossing legs in both sitting and standing and to sleep in supine with a pillow under their knees or in sidelying with a pillow between their legs.

### Should pharmacologic agents be utilized in the setting of GTPS?

*Consensus statement* **: The indications for the use of pharmacological agents vary and will depend on patient demographics and history, as well as physician preferred practice pattern.**

CSIs and platelet-rich plasma (PRP) injections are widely utilized conservative treatment interventions for GTPS. CSIs are primarily utilized to reduce pain and improve function. Lievense et al. [32] reported that patients who received a CSI were 2.7 times more likely to achieve recovery in 5 years compared to those who did not receive a CSI. Similarly, Shbeeb et al. [33] reported that 61.3% of patients receiving a CSI reported a reduction in pain severity and functional limitations at 26 weeks. Conversely, Brinks et al. [34] evaluated the effect of CSI compared to usual care in the setting of GTPS. At 3 months, CSI demonstrated a clinically relevant effect in regard to recovery, pain at rest, and pain with activity; however, these effects were no longer present at 12 months [34]. It should be noted that, however, the diagnosis of GTPS was identified through palpation of the greater trochanter, which has a low specificity for identifying gluteal tendinopathy when utilized alone [7]. No differences in outcomes are observed with the use of a fluoroscopic-guided injection into the trochanteric bursae [35]. CSIs targeting the greater trochanteric bursae carry a low risk for side effects; however, prolonged steroid use can result in possible tendon degeneration and rupture. Additionally, studies have demonstrated inhibitory effects of tendon repair and delayed tendon sheath healing [10, 36–38]. In other areas, such as the common extensor tendon of the elbow, outcomes following a CSI are conflicting and sometimes met with less favourable results [39].

With concern for involvement of the GMed or GMin tendons, the use of PRP may be advised as PRP offers a minimally invasive technique to promote tissue healing through a high dose of platelet-derived growth factors. These growth factors help to activate the healing cascade and reverse the degenerative process [40–44]. Three randomized control trials investigated the use of PRP injections in the setting of GTPS with conflicting results [45–47]. Fitzpatrick et al. [45] and Jacobson et al. [47] found superior clinical outcomes with the use of PRP compared to CSI in patients with GTPS, whereas Riberio et al. [46] found no significant differences in pain or function. However, it should be noted that these studies utilized differing injection volumes, spinning protocols and compensations, which may affect the efficacy and generalizability of the study results. A recent systematic review reported improved outcomes for PRP at 2 years compared to a CSI as measured by the Harris Hip Score (HHS) [48]. Additionally, Fitzpatrick et al. [49] reported a sustained benefit through 2 years following PRP, whereas the benefits of a CSI were not observed beyond 24 weeks. Other emerging pharmacological agents include the use of menopausal hormone therapy, which has been shown to be effective in women with a body mass index (BMI) <25 in conjunction with any form of exercise or education [50].

### Should physiotherapy be utilized following the use pharmacologic agents?

***Consensus statement*** **: Physiotherapy management, including gait training, soft tissue mobilization, and therapeutic exercises, should be utilized following the use of pharmacological agents.**

Physiotherapy should be utilized after both CSI and PRP injections as these conservative interventions do not improve the modifiable factors, including hip abductor weakness, loss of pelvic control in the frontal plane and iliotibial band tightness, linked to the development of GTPS [25, 26]. While there is no evidence-based timeline for when to resume the activity following a CSI, the panel recommends resuming physiotherapy when tolerated by the patient. Following a PRP injection, a longer period of rest is recommended to not interfere with the healing cascade. In both cases, physiotherapy should begin with gentle isometrics, pain-free stretching that avoids compressive forces through the lateral gluteal tendons [25] and non-weight bearing and weight bearing lumbopelvic and lower extremity neuromuscular control activities. When tolerated, eccentric hip abductor strengthening and dynamic single leg activities focusing on pelvic control in the frontal plane should be prescribed. Patient education should include minimizing positions that increase load and compression through the lateral hip.

### What are the indications for surgical management of GTPS?

*Consensus statement*: The indications for surgical management vary based on individual patient characteristics and surgeon-specific criteria. These findings may include imaging evidence of a GMed tear, failed previous appropriate conservative management, a severe gait deviation, the inability to abduct hip against gravity, and tissue quality.

Although 60–90% of individuals with GTPS will respond positively to conservative management, those with prolonged pain and dysfunction despite appropriate conservative interventions may require surgical intervention [51, 52]. Multiple low-level studies report improved outcomes following the surgical management of gluteal tears [40, 53–58]; however, there is limited evidence supporting specific objective findings that support success or failure of surgical intervention for GTPS. Chandrasekaran et al. [59] found that individuals presenting with a GMed tear on initial evaluation were more likely to fail conservative management and require surgical intervention if they demonstrated a reduction in hip abduction power and the presence of a gait deviation. Our expert panel did not reach consensus regarding a cluster of findings that would indicate need for surgical intervention. Therefore, decisions regarding surgical intervention need to be made on an individual basis and may include factors such as imaging evidence of a GMed tear, failed previous appropriate conservative management including physiotherapy and injections, presence of a severe gait deviation, a severe strength deficit of the hip abductors and tissue quality.

### Are there prognostic indicators that would negatively affect outcomes following surgical management of GTPS?

Consensus: Poor prognostic indicators for surgical intervention for GTPS include age, BMI, size of tear, tissue quality, tobacco use, diabetes, and low physical function.

Both open and endoscopic repairs of the hip abductors have shown improved functional outcomes with fewer complications reported with the endoscopic technique [60]. Retears were found in 3.4% and 3.1% of individuals following open and endoscopic repairs, respectively [60]. The panelists agreed that certain patient characteristics may lead to poorer outcomes following the surgical intervention. In the setting of rotator cuff repairs, advanced age, BMI, tobacco use and diabetes have been linked to poor healing and worse outcomes and larger tear size was identified as an independent risk factor for poor healing [61–64]. It can be assumed that the same factors would have an effect on healing of the hip abductors. Additionally, tissue quality may affect postoperative healing. A recent systematic review and meta-analysis found that high-grade fatty infiltration of the hip abductors, indicating poor tissue quality, resulted in less improvement on the HHS and the Modified HHS [65]. When considering surgical intervention for the management of GTPS, these patient characteristics should be taken into account in both surgical planning and preoperative patient education.

### Should physiotherapy be utilized following surgical management of GTPS?

*Consensus statement*: Physiotherapy management, including gait training, soft tissue mobilization, and therapeutic exercises, as outlined in Table II, should be utilized following a period of tissue healing after surgical management of GTPS.

**Table II.** Panelist recommendations for rehabilitation guidelines following surgical management of GTPS

Gluteal Repair Physiotherapy Guidelines	
*Phase I: Immediate Postoperative Phase* (*Weeks 1–6)*	
*Goals:* Protect healing tissues Reduce postoperative pain and inflammation Normalized gait pattern with an appropriate assistive device	*Precautions:* Weight-bearing (WB) : Foot flat weight bearing 20% body weight Range of motion: Hip flexion limited to 90° Hip external rotation limited to 20° Hip adduction limited to neutral Active, against gravity hip abduction contraindicated until postoperative week 8 Active long lever hip flexion contraindicated until week 12
*Therapeutic interventions:* Ankle pumps, hamstring, and quadriceps setting Submaximal isometrics of hip adductors, hip extensors and lower abdominals Cryotherapy and compression for inflammation and oedema control Aqua therapy for gait training upon healing of surgical incision	
*Phase II: Early postoperative phase* (*Weeks 6–8*)	
*Goals:* Gentle progression of ROM Continue protecting healing soft tissues Limit irritation of hip flexors and hip abductors through slow, gentle progression	*Precautions:* Weight-bearing: progress to FWB as tolerated Active, against gravity hip abduction contraindicated until postoperative week 8 Active long lever hip flexion contraindicated until week 12
*Physical therapy:* Initiate upright stationary bike with no resistance Submaximal isometrics in all directions, including hip abductors in a gravity eliminated position Gradual loading of iliopsoas tendon is critical to avoid tendonitis Short lever active (AROM) and active assistive (AAROM) for hip ROM Lumbopelvic neuromuscular control exercises in supine	
*Phase III: strengthening phase (Weeks 8–12)*	
*Goals:* Near full, symmetrical ROM Improve hip and core strength and neuromuscular control Gradual WB progression (normalized gait pattern and physician clearance required for for weaning from assistive device)	*Precautions:* Monitor for symptoms of intra- and extra-articular irritation with exercise and WB progression Avoid premature weaning from the assistive device Active long lever hip flexion contraindicated until week 12
*Physical therapy:* Gradual progression of functional ROM Upright bike with progressive resistance Progress from hip abductor isometrics in gravity eliminated positions to isotonic in positions of gravity as tolerated Introduce elliptical between 8 and 10 weeks as tolerated Initiate closed chain strengthening progression with focus on single leg pelvic control as tolerated Progress lumbopelvic stabilization and postural control exercises	
*Phase IV: return to low-level impact* (*weeks 12–16)*	
*Goals:* Tolerance of running and agility drills with appropriate lumbopelvic and lower extremity control	*Precautions:* Avoid provocation of symptoms with progression of exercise No jumping, hopping, cutting/pivoting
*Physical therapy:* Initiate running and agility progressions with emphasis on dynamic control of lower extremity and pelvis Continue high-level strength and control exercise with focus on single leg pelvic control	
*Phase V: return to full participation in sports* (*weeks 16+)*	
*Goals:* Tolerance of jumping, hopping, cutting/pivoting drills with appropriate lumbopelvic and lower extremity control Return to full participation in sports	*Precautions:* Avoid provocation of symptoms with progression of exercise
*Physical therapy:* Initiate jumping and hopping progression with emphasis on dynamic control of lower extremity and pelvis Sport-specific cutting and pivoting drills with emphasis on dynamic control of lower extremity and pelvis	

Following surgical repair of the hip abductors, postoperative rehabilitation should be utilized to restore tissue mobility, lumbopelvic and posterolateral hip strength and appropriate control of the pelvis in the frontal plane. However, there is limited evidence supporting a specific physiotherapy protocol following surgical repair of the hip abductors. Stanton et al. [66] recommended 12 weeks of restricted weight bearing as well as limitations in hip flexion, hip abduction and hip internal rotation for 6–8 weeks in individuals with an open repair of the GMed and GMin. Following endoscopic repair of GMed tears, Thaunat et al. [67] restricted active physiotherapy for 6 weeks at which time a gradual weight bearing and range of motion should be utilized. Others have recommended a three-phase protocol with 6 weeks of protected weight bearing and gentle passive range of motion, followed by 6 weeks of progressive weight bearing and hip strengthening, followed by a return to activity progression [55, 68, 69]. Progression should emphasize pain-free range of motion and submaximal isometrics followed by concentric and eccentric loading and finally functional activities (Table II). Similar to the conservative management of GTPS, physiotherapy management should focus on minimizing factors that increase compressive load through the lateral aspect of the hip and restore appropriate pelvic control. A graded approach to therapeutic exercise following surgical intervention is crucial to allow for improvements in strength while minimizing musculotendinous overload and joint irritation. Panelists agree that rehabilitation guidelines should always be guided by clinical reasoning, prior level of function and individual patient goals.

### Limitations

There are several limitations to our current study. There is limited high-quality research supporting non-operative and operative management of GTPS. Therefore, initial study questions were generated by expert opinion, which could result in bias. To minimize resultant bias, the authors recruited a diverse, international panel with expertise in the management of GTPS. Additionally, panelists completed all survey rounds on-line, which does not allow for clarification or open discussion regarding item questions.

## CONCLUSION

Although physiotherapy management of GTPS is important to help patients return to the prior level of function, there is paucity of research supporting specific guidelines. This international consensus statement provides guidelines for physiotherapy management of patients presenting with GTPS according to the current knowledge and expertise regarding the following: (i) evaluation of GTPS, (ii) non-surgical physiotherapy management, (iii) use of CSIs and orthobiologics and (iv) surgical indications and postoperative physiotherapy management.

## Data Availability

The data underlying this article will be made upon request to the corresponding author.
